# Natural pigments from the endophyte *Aspergillus westerdijkiae* and evaluation of their bioactivities

**DOI:** 10.1186/s12934-025-02905-8

**Published:** 2025-12-31

**Authors:** Abirami Baskaran, Andreas Wasilewicz, Judith M. Rollinger, Joanna Grzelczyk, Ilona Gałązka-Czarnecka, Grażyna Budryn, Tomasz Strzała, Tyler N. Graf, Nicholas H. Oberlies, Filip Boratyński, El-Sayed R. El-Sayed

**Affiliations:** 1 Department of Food Chemistry and Biocatalysis, Wrocław University of Environmental and Life Sciences, Norwida 25, Wrocław, 50-375 Poland; 2 Division of Pharmacognosy, Department of Pharmaceutical Sciences, University of Vienna, Josef-Holaubek-Platz 2, Vienna, 1090 Austria; 3 Institute of Food Technology and Analysis, Faculty of Biotechnology and Food Sciences, Lodz University of Technology, Lodz, 90-537 Poland; 4 Department of Genetics, Wroclaw University of Environmental and Life Sciences, Kożuchowska 7, Wrocław, 51-631 Poland; 5 Department of Chemistry and Biochemistry, University of North Carolina at Greensboro, Greensboro, NC 27402 USA; 6 Plant Research Department, Nuclear Research Center, Egyptian Atomic Energy Authority, Cairo, Egypt

**Keywords:** Natural pigments, Monoamine oxidase, Cholinesterase inhibitors, PPAR-γ, Fungal endophytes

## Abstract

**Background:**

The growing consumer preference for natural and sustainable products has heightened interest in biopigments across pharmaceutical, cosmetic, and food industries. In this study, we investigate endophytic fungi as a viable and eco-friendly source for the production of bioactive natural pigments.

**Methods and results:**

A promising strain, *Aspergillus westerdijkiae* 17P, was isolated from *Betula pendula* and assessed for its pigment-producing potential and associated bioactivities. The biomass extract was fractionated, and the resulting components were evaluated for antimicrobial, antioxidant, anticancer, neuroprotective, and peroxisome proliferator-activated receptor gamma (PPAR-γ) agonist activities. Among the fractions, 17P2 exhibited broad-spectrum antimicrobial effects, notable antioxidant activity (83% DPPH radical scavenging at 1000 mg/mL), and cytotoxicity against MCF-7 and HepG2 cancer cell lines, with IC₅₀ values of 250 mg/mL. Isothermal titration calorimetry (ITC) demonstrated strong binding affinities of 17P2 to acetylcholinesterase (Kd = 1.63 µM) and butyrylcholinesterase (Kd = 0.03 µM), indicating potential anti-Alzheimer’s properties. Additionally, significant interactions with monoamine oxidase A and PPAR-γ suggest possible antidepressant and antidiabetic applications. Four major pigment fractions (17P1–17P4) were purified and structurally characterized using UHPLC-MS and NMR, revealing key metabolites such as aluminium and iron aspergillic acid complexes, penicillic acid, and preussin. Notably, gamma irradiation at 2000 Gy significantly enhanced the red, yellow, and orange pigments yield compared to the non-irradiated control cultures.

**Conclusions:**

Collectively, these findings position *A. westerdijkiae* 17P as a valuable and versatile biotechnological resource for the sustainable production of multifunctional fungal pigments with potential industrial and therapeutic applications.

## Introduction

 Colors play a fascinating part in human life profoundly influencing emotions and preferences. Anciently, a rich variety of dyes and pigments originating from natural fruit and vegetable extracts were utilized for architectural, decorative and preservative purposes [1]. Even though the previous centuries saw applications of synthetic colorants in textile, food, cosmetic and pharmaceutical industries, their toxicity and non-environment friendly manufacturing practices have started limiting their use and enhanced the search for bio-pigments from natural sources [2]. Colorants sourced from algae, insects, plants and animals, face limitations such as low yields, seasonal availability, deforestation, instability, and insolubility [2]. However, microbial pigments offer significant advantages like consistent supply, greater stability, cost efficiency, and higher yields [3]. Fungi are a remarkable source of natural pigments often displaying a wide spectrum of colors with improved solubility and stability. Notably, individual fungal species can biosynthesize diverse pigments with distinct properties [4, 5]. Fungal mycelium releases these pigments as secondary metabolites, generally, as a response to environmental stress or nutrient limitation [6]. These fungal pigments also possess multifaceted biological activity such as cellular differentiation, metal transport, complex interactions with other organisms through symbiosis and competition, even providing defense against predators and insects [7], all of which generated interest in the pharmaceutical field, documenting their potential as antiviral, antifungal, antibacterial, antioxidant, anti-inflammatory, antitumor, anti-Alzheimer’s disease, anti-atherosclerotic, anti-obesity, immunosuppressive, nematocidal, and cytotoxic agents [8].

Endophytic fungi comprise a ubiquitous and diverse microbial group inhabiting all plant species across a wide range of climatic and ecological zones [9]. Isolated from virtually every plant type, from trees, shrubs, herbs to ferns and marine plants, their diversity is significantly influenced by host plant genotype and physiology, environmental factors, human activities and growth season [10]. This diversity extends to differences among host plants within the same geographical area and even within different parts of a single plant [11]. Notably, tropical and subtropical regions exhibit a significantly higher diversity of fungal endophytes compared to other areas [12, 13]. The co-evolution between fungal endophytes and their plants results in bioactive metabolites production that provide multiple benefits such as stimulating plant growth, triggering defense mechanisms against pathogens and aid in tolerating drought and salt stresses [14–16]. These endophytes enhance plant defence by producing antimicrobial metabolites and enzymes, competing with pathogens, and activating host immune pathways [14]. They also improve abiotic stress tolerance by boosting antioxidant enzyme activity, osmolyte accumulation, ion homeostasis, and hormone modulation, thereby enhancing water balance, photosynthesis, and overall resilience to drought and salinity stresses [10, 14]. For their ability to quantitatively increase the production of secondary metabolites in host plants and their extensive distribution and diversity, these endophytic fungi are often considered superior to other fungi [17].

In this study, pigments from the endophyte *Aspergillus westerdijkiae* 17P were separated and their bioactive properties including antimicrobial, cytotoxic, antioxidant effects, monoamine oxidase A, acetylcholinesterase, and butyrylcholinesterase inhibiton, and peroxisome proliferator-activated receptor gamma agonistic potential were analysed. The impact of gamma irradiation on pigments production was evaluated. Chemical characterization of pigments fractions was also studied.

## Materials and methods

### Isolation of endophytic fungi

*Betula pendula* samples were collected from Mokrzański forest, Poland. The plant was identified with the help of Dr. Katarzyna Patejuk, Department of Plant Protection, Wrocław University of Environmental and Life Sciences. Healthy plant parts were collected, stored in ice box and transported to the laboratory. The isolation of fungal endophytes was accomplished according to previous study [18]. The samples were cut into small pieces, surface-sterilized by dipping in 70% ethanol for 1 min, followed by 0.1% HgCl_2_ for 1 min, and then rinsed in sterile distilled water before being dried on sterile filter paper. The sterilized plant fragments were then fragmented into smaller pieces and aseptically transferred onto potato-dextrose agar plates (supplemented with streptomycin and tetracycline) and incubated at 25 °C. The inoculated plates were checked daily and the isolated cultures were sub-cultured for purity and stored at − 4 °C in glycerol (15%) as a suspension of spores and mycelia.

### Identification of the fungal strain 17P

Among the isolated fungi, a promising strain, designated as 17P, was isolated from a twig of *Betula pendula*. The 17P strain was chosen based on its pigments producing ability. The fungus was identified using cultural, morphological and molecular methods. Cultural characteristics were studied on Malt Extract agar (MEA), Yeast Extract Sucrose agar (YES), Czapek–Yeast Autolysate agar (CYA), and Potato-dextrose agar (PDA) at 30 °C for 10 days.

The identity of the fungus was confirmed by molecular characterization techniques. DNA was extracted from fungal cultures using the Genomic Mini AX Yeast kit (A&A Biotechnology, Gdańsk, Poland) following the manufacturer’s protocol. The DNA concentration was then quantified using the Qubit 4.0 fluorometer. Amplification of the ITS region was performed using the ITS4 (TCCTCCGCTTATTGATATGC) and ITS5 (GGAAGTAAAAGTCGTAACAAGG) primers under the following thermal cycling conditions: an initial denaturation at 95 °C for 2 min; 35 cycles of 95 °C for 60 s, 55 °C for 60 s, and 72 °C for 90 s; followed by a final elongation at 72 °C for 10 min. PCR products were visualized via electrophoresis on a 1% agarose gel and subsequently purified using NucleoMag magnetic beads (Macherey-Nagel). Sequencing was carried out using the Oxford Nanopore Technology (ONT) MinION platform, employing the SQK-NBD114.96 barcoding kit and a FLO-MIN114 flow cell. Library preparation followed ONT’s guidelines for barcoded samples. Raw sequence data in POD5 format were basecalled using the Dorado basecaller (https://github.com/nanoporetech/dorado) with the super accurate (SUP) model. FastQC (https://www.bioinformatics.babraham.ac.uk/projects/fastqc/) was used for quality assessment, and reads were trimmed to the appropriate length with a minimum phred score of 12 using Chopper [19]. Consensus sequences were generated using Scaffold Builder [20]. Initial species identification was conducted through the MycoBank database. Sequences from the study were then incorporated into a phylogenetic analysis alongside ITS sequences of known species obtained from NCBI, including outgroup. Phylogenetic relationships were inferred using the Bayesian framework implemented in MrBayes 3.2.7a [21], with the mixed model selection. Trees were sampled every 100th generation over 10,000,000 MCMC generations, and the consensus tree was constructed from samples with an average standard deviation of split frequencies well below 0.01.

### Preparation and extraction of fungal biomass

Spore suspensions were obtained from 7-day-old fungal cultures. Spore concentrations were determined and standardized to 10^4^ spores·mL⁻¹ using a hemocytometer. Under aseptic conditions, 1 mL of the standardized spore suspension was inoculated into 250 mL Erlenmeyer flasks containing 50 mL of potato dextrose (PD) broth. The flasks were incubated at 30 °C under static conditions for 14 days.

Following incubation, cultures were filtered through Whatman No. 1 filter paper to separate the culture filtrate from the fungal biomass. The recovered mycelial biomass was homogenized using a mortar and pestle until a uniform consistency was achieved. Each homogenized sample was extracted with 25 mL of a chloroform–methanol mixture (9:1, v/v). The extraction mixtures were sonicated for 1 h at 20 kHz and 20 °C in an ultrasonic bath.

Subsequently, the organic phase was separated using a separatory funnel and dried over anhydrous sodium sulfate. The solvent was removed under reduced pressure using a rotary vacuum evaporator to yield dry extracts. These extracts were reconstituted in a methanol–dimethyl sulfoxide mixture (2:1, v/v) for further analysis.

### Fractionation and chromatographic separation of the extract

The fungal extract was adsorbed onto silica gel (Silica Gel 60, 0.040–0.063 mm particle size; Merck, Germany) and the solvent was removed under reduced pressure using a rotary vacuum evaporator. A chromatographic column was packed to three-quarters of its volume with silica gel, after which the silica–extract mixture was added.

Flash chromatography was performed using a puriFlash^®^ XS520 Plus system (Interchim SA, France) equipped with a silica-based column (SIHP-JP, F0012). Elution was carried out using a gradient system of n-hexane and ethyl acetate. The separation commenced with 100% n-hexane, followed by stepwise increases in the ethyl acetate concentration (1%, 2%, and finally 5%, v/v), thereby increasing the polarity of the mobile phase. This process resulted in the isolation of four pigment-containing fractions, designated as 17P1, 17P2, 17P3, and 17P4.

### Testing bioactivities of the separated fractions

#### Antimicrobial activity

The antimicrobial activity of the isolated pigment fractions (17P1–17P4) was assessed using the agar well diffusion method [22]. Antibacterial efficacy was evaluated against human pathogenic strains, including *Staphylococcus aureus* ATCC6538, *Pseudomonas aeruginosa* ATCC9027, *Klebsiella pneumoniae* ATCC13883, and *Escherichia coli* ATCC11229, while antifungal activity was tested against *Fusarium oxysporum* EUM37, *Alternaria alternata* EUM108 (phytopathogens), *Candida albicans* ATCC10231, and *Aspergillus brasiliensis* ATCC16404 (human pathogens). The positive control for antibacterial assays was a mixture of amoxicillin and clavulanic acid (500 µg/mL), and for antifungal assays, nystatin (100 µg/mL) was used. The solvent mixture (MeOH: DMSO, 2:1 v/v) served as the negative control.

Bacterial cultures were standardized to 0.5 McFarland turbidity using spectrophotometric measurement at 600 nm (A₆₀₀ = 0.08–0.10). Fungal spore suspensions were adjusted to 1 × 10⁶ spores/mL using a hemocytometer. One hundred microliters of each microbial suspension were inoculated onto appropriate media: Mueller–Hinton Agar for bacteria, Potato Dextrose Agar for fungi, and Sabouraud Dextrose Agar for *Candida*. Agar wells were created and filled with 50 µL of each fungal extract. Plates inoculated with *E. coli*, *S. aureus*, and *C. albicans* were incubated at 37 °C for 24 h, while those containing *F. oxysporum* and *A. brasiliensis* were incubated at 30 °C for 5 days. Zones of inhibition (ZOI) were measured in millimeters.

#### DPPH scavenging assay

The antioxidant potential of the pigment fractions was evaluated using the DPPH (1,1-diphenyl-2-picrylhydrazyl) radical scavenging assay [23]. A 120 µL aliquot of 100 µM DPPH in methanol was mixed with 80 µL of each pigment extract. Ascorbic acid (10 mM) served as the positive control, while the solvent mixture (DMSO: MeOH, 1:2 v/v) served as the blank. After 15 min of incubation in the dark at room temperature, the absorbance was measured at 517 nm.

#### Cytotoxic activity assay

The cytotoxic effects of pigment fractions (17P1–17P4) were evaluated against three human cancer cell lines: breast adenocarcinoma (MCF-7), and lung carcinoma (A549), along with a non-cancerous human fibroblast cell line (Hfb-4). All cell lines were procured from ATCC and cultured in Dulbecco’s Modified Eagle Medium (DMEM) supplemented with 10% fetal bovine serum, 1% penicillin-streptomycin, and L-glutamine (Sigma-Aldrich). All cells were tested for Mycoplasma contamination before experiments. Cytotoxicity of the pigments fractions 17P1, 17P2, 17P3 and 17P4 was evaluated against the aforementioned cell lines using the MTT assay according to the method described by Van de Loosdrecht and co-workers [24] with slight modifications. Cells were seeded at 1 × 10⁴ cells/well in 96-well plates and incubated at 37 °C in a humidified 5% CO₂ atmosphere. Cells were treated with 20 µL of each pigment fraction for 24 h. Untreated control wells received 0.1% DMSO. After treatment, 10 µL of MTT (5 mg/mL) was added to each well, followed by incubation for 4 h. The resulting formazan crystals were solubilized in 100 µL of DMSO. Absorbance was read at 570 nm using a TECAN SunRise microplate reader. Cytotoxicity was calculated using:

$${\textrm{Inhibition}} \ (\%) = [100 - ({\textrm{A570 of treated cells}}\, / \,{\textrm{A570 of control cells}})] \times 100$$

#### AChE and BChE inhibitory potentials

Enzymatic inhibition studies were conducted using acetylcholinesterase (AChE) from *Electrophorus electricus* and butyrylcholinesterase (BChE) from horse serum (Sigma-Aldrich). The isothermal titration calorimetry (ITC) assay was performed using a MicroCal PEAQ-ITC200 instrument (Malvern Instruments, UK). The sample cell (0.2 mL) was loaded with a 20 µM solution of either AChE or BChE in methanol. Pigment fractions (10 mM in methanol) were injected in 2 µL increments. Binding thermodynamics were recorded at 36 °C under constant stirring. Control titrations (methanol only) were subtracted from enzyme–ligand interactions. Binding parameters including dissociation constant (Kd), association constant (Ka), enthalpy change (ΔH), entropy change (ΔS), and Gibbs free energy change (ΔG) were calculated using single-site binding models via the MicroCal PEAQ-ITC software [25]. Competitive inhibition was also assessed in the presence of acetylcholine (ACh).

#### MAO-A inhibitory potential

MAO-A inhibitory potential was evaluated under similar ITC conditions with modifications. A 10 µM solution of human recombinant MAO-A was titrated with 2 mM pigment fractions and/or serotonin (5-HT, positive control). A total of 11 injections were performed over 30 min [26].

#### PPAR-γ agonist potential

GST-tagged human PPAR-γ ligand-binding domain (residues 204–477; 50 µg/mL) and the natural ligand 15-deoxy-Δ¹²,¹⁴-prostaglandin J2 (≥ 95%) were obtained from Sigma-Aldrich. ITC analysis was conducted as described for BChE, with a 10 µM solution of PPAR-γ used as the target and 20 µM pigment fractions or prostaglandin J2 as ligands. The measurements were carried out at 36.6 °C, with 11 injections over 30 min [27].

### Effect of ^60^Co gamma irradiation on pigments production

Spore suspensions of *Aspergillus westerdijkiae* 17P were subjected to γ-irradiation at doses of 250, 500, 1000, 2000, 4000, 8000, and 16,000 Gy using a ⁶⁰Co gamma irradiator (MC20, Russia; dose rate 311.88 Gy/h). Post-irradiation, suspensions were incubated in the dark overnight, then 1 mL was inoculated into 50 mL of PD broth (pH 6.0) and cultured at 30 °C for 14 days.

After incubation, biomass was harvested, extracted as previously described, and pigment concentrations were determined spectrophotometrically at 410 nm (yellow), 470 nm (orange), and 510 nm (red). Fungal biomass (g·L⁻¹) was measured by drying to constant weight at 50 °C.

Spore survival rates were assessed by plating 100 µL of irradiated spore suspensions on PDA, incubating at 25 °C for 5 days, and counting colony-forming units. The survival rate was expressed relative to unirradiated controls (considered 100%).

### Chemical characterization

The whole biomass extract of the fungus was first analyzed via LC-MS dereplication using a database of over 700 fungal metabolites using procedures that have been described previously [28, 29].

Second, UHPLC-ELSD (Ultra High-Performance Liquid Chromatography-Evaporative Light Scattering Detector) and UHPLC-UV-MS analyses were performed on a Waters Acquity UPLC H-Class system consisting of a fraction manager, column manager, quaternary solvent manager, PDA detector, ELS detector, isocratic solvent manager and a single quadrupole mass detector (Acquity QDa) equipped with an ESI source. A BEH C_18_ column (1.7 μm, 2.1 × 100 mm, Waters) was used as stationary phase; water + 0,1% formic acid (A) and acetonitrile + 0,1% formic acid (B) were used as mobile phase. The column temperature was set to 40 °C, flow rate of 0.3 was used and the following gradient was applied: 5% B at 0.0 min, from 5% − 98% B in 5 min, 98% B for 10 min. Mass detection was performed in positive (cone voltage: 15 V, capillary voltage 0.8 kV) and negative (cone voltage: 30 V, capillary voltage: 0.8 kV) mode from 200 to 1200 Da. To increase ionization, a mixture of water: methanol (9:1) + 10 mM ammonium formate was used as make-up solvent. The instrument was controlled by Empower 3.

Size exclusion chromatography was conducted for the separation of 17P2, using Sephadex LH-20 (GE Healthcare Bio-Sciences AB, Uppsala, Sweden) as stationary phase and MeOH as mobile phase (column dimensions: 100 cm × 2 cm). The fractions were collected in a time-dependent manner (3 min/tube) and analyzed by thin-layer chromatography (TLC) using Merck silica gel 60 PF254 plates as stationary phase and ethyl acetate as mobile phase. Detection was performed at visible light and UV366. Based on the TLC fingerprints, the collected tubes were pooled into 5 fractions (17P2_I-V).

1D and 2D NMR data of 17P4 were recorded using a Bruker UltraShield 500 MHz NMR spectrometer equipped with a TCI Prodigy CryoProbe (5 mm), an AVANCE III HD console and a SampleJet. The fraction was measured at 296 K in MeOD referenced to the residual non-deuterated solvent signals (δH 3.31 ppm; δC 49.00 ppm). The resonance frequency for ^1^H NMR was 500.19 MHz and for ^13^C NMR 125.77 MHz. Standard 1D (1H) and gradient-enhanced 2D experiments, i.e. HSQC, and HMBC, were used as supplied by the manufacturer. The raw NMR data were processed using Topsin 4.3.0.

Penicillic acid (5): identified in 17P4. ^1^H NMR (MeOD, 500 MHz): 5.47 (1H, m), 5.28 (1H, s), 5.24 (1H, m), 3.89 (3 H, s); ^13^C NMR (MeOD, 125 MHz): 176.2 (qC), 173.5 (qC), 117.4 (CH_2_), 91.1 (CH), 59.7 (OCH_3_), 17.4 (CH_3_); ESI-MS: *m/z* 171.13 [M + H]^+^.

Preussin (6): identified in 17P4. ^1^H NMR (MeOD, 500 MHz): 7.34 (2Hs, m, 2x H-3’), 7.32 (2Hs, m, 2x H-2’), 7.24 (1H, m, H-4’), 4.03 (1H, m, H-3), 3.12 (1H, dd, H-1a), 2.96 (1H, dd, H-1b), 3.03 (1H, m, H-2), 2.92 (1H, m, H-5), 2.67 (3Hs, s, NCH_3_), 2.36 (1H, m, H-4a), 1.63 (1H, m, H-4b), 0.91 (3Hs, t, H-14); ^13^C NMR (MeOD, 125 MHz): 139.3 (CH, C-1’), 130.3 (2x CH, C-3’), 129.7 (2x CH, C-2’), 127.7 (CH, C-4’), 75.3 (CH, C-2), 69.8 (CH, C-3), 69.3 (CH, C-5), 39.3 (CH_2_, C-4), 38.8 (NH, NCH_3_), 32.5 (CH_2_, C-1), 14.4 (CH_3_, C-14); ESI-MS: 318.27 [M + H]^+^.

### Statistics

Isothermal titration calorimetry (ITC) data were analyzed based on the mean values obtained from three independent replicates (*n* = 4), with results expressed as mean ± standard deviation (SD). Statistical significance was assessed using one-way analysis of variance (ANOVA) performed in SPSS software (version 22.0; IBM Corp., NY, USA). Differences were considered statistically significant at *P* < 0.05.

## Results and discussion

### Identification of the 17P fungus

**Fig. 1:**
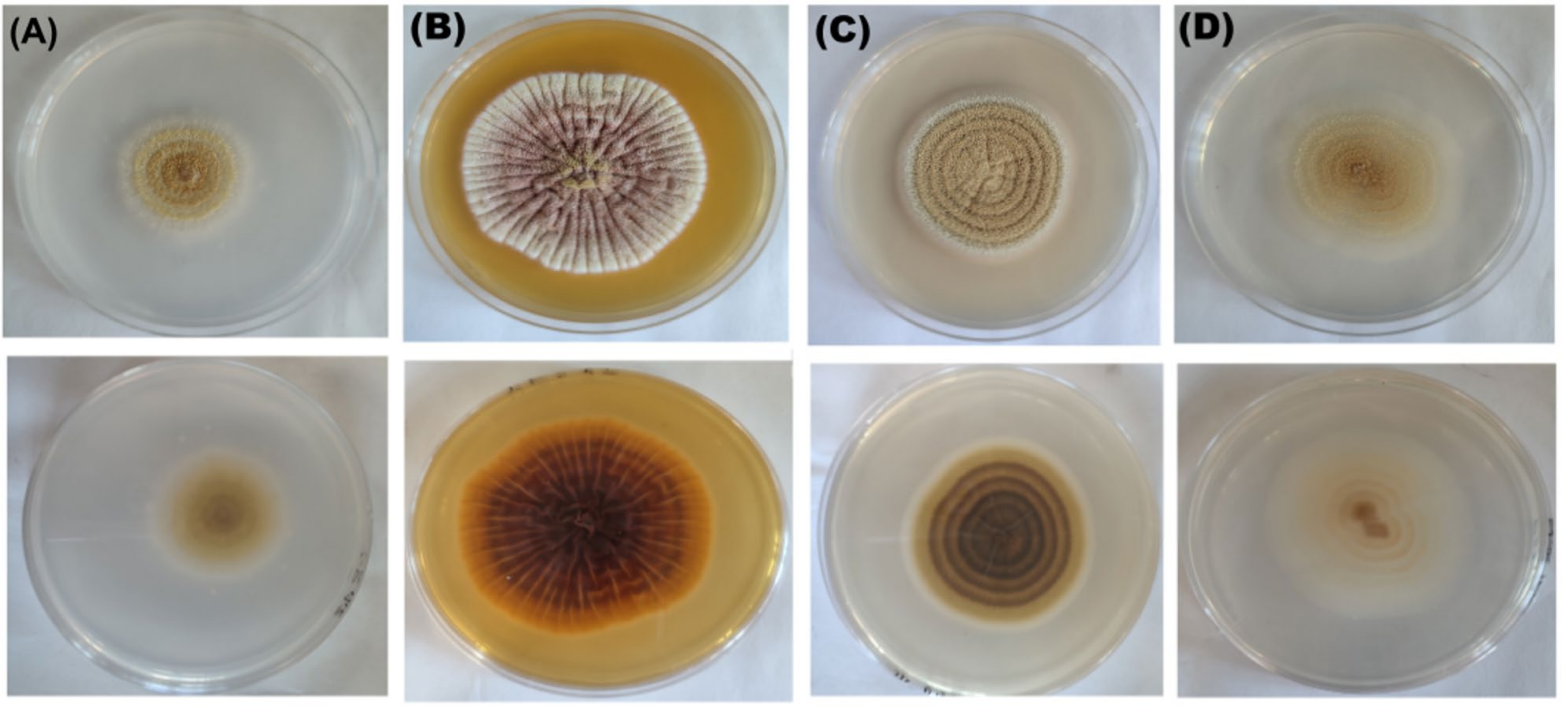
Morphological characteristics of the 17P strain. Colonial growth was observed on Czapek–Yeast Autolysate agar **A**, yeast-sucrose agar **B**, malt extract agar **C** and potato-dextrose agar **D**. The plate cultures in the top shows a front view of the growth and the plate cultures in the bottom shows a reverse view of the growth after incubation for 10 days at 30 °C

**Fig. 2:**
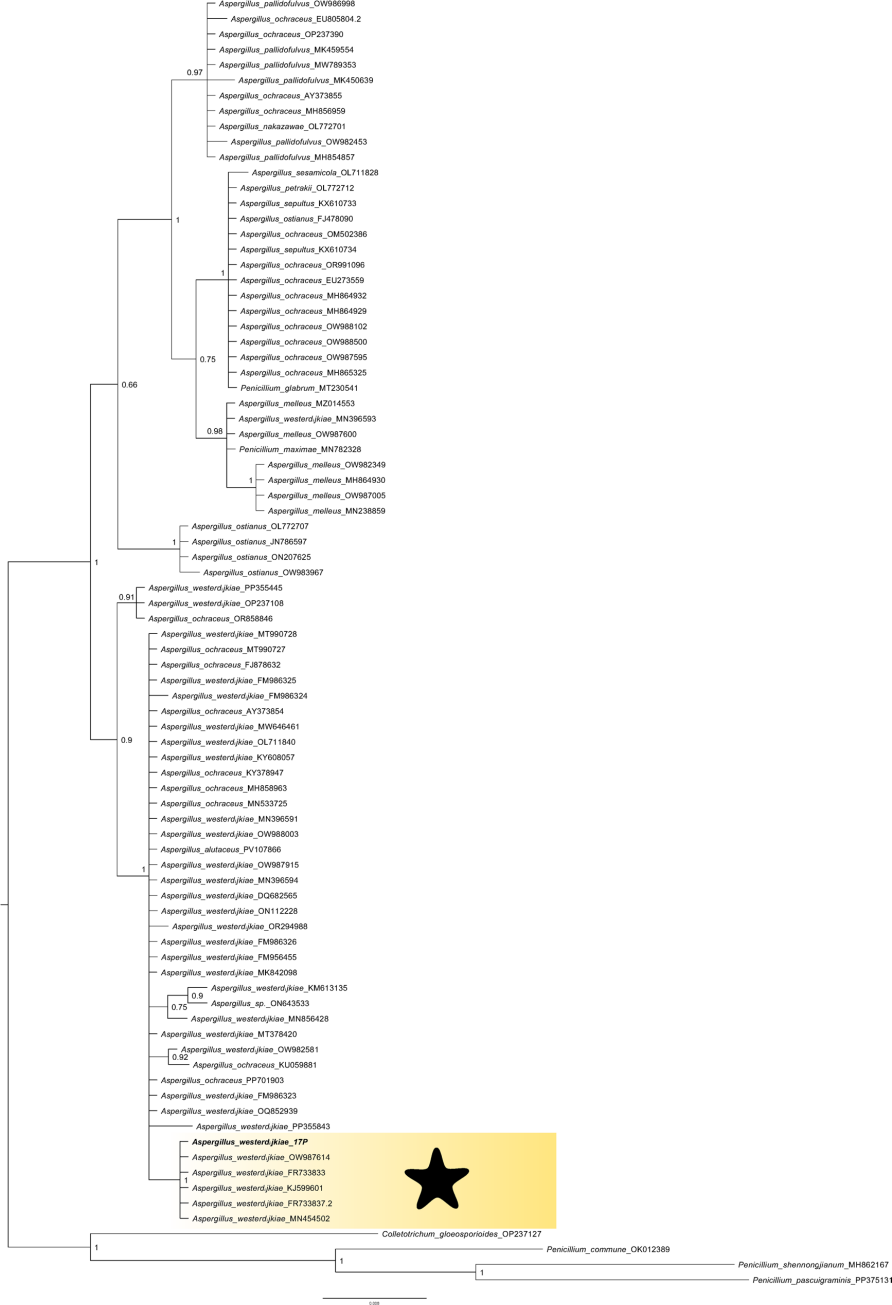
Bayesian phylogenetic tree of the ITS sequence from 17P strain (this study) and sequences possessed form NCBI (accession numbers of all used NCBI sequences are showed on the tree). Numbers along the nodes are the posterior probabilities of the nodes. *Aspergillus westerdijkiae* clade is shaded and marked with a star shape. The 17P sample analyzed in this study is bolded

Figure 1 shows the colony morphology of the 17P strain maintained on the CYA (Fig. 1a), YES (Fig. 1b), MEA (Fig. 1c), and PDA (Fig. 1d). Colony characters: CYA 30 °C, 7 d: Colony surface floccose; mycelial areas white to greyish yellow; sporulation pale yellow to light yellow; reverse greyish yellow. YES 30 °C, 7 d: Colony surface floccose; mycelial areas pinkish and white near margin; sporulation greyish yellow; reverse dark brown. MEA 30 °C, 7 d: Colony surface floccose; mycelial areas brown with white margin; sporulation light yellow; reverse dark brown. PDA 30 °C, 7 d: Colony surface floccose; mycelial areas pale yellow; sporulation greyish yellow; reverse pale yellow, yellowish and white near margin. These characteristics are identical with those reviewed by previous reports [30, 31] concerning identifications of *Aspergillus westerdijkiae*. For analyzed 17P sample, 1,000 high-quality reads (post-trimming) were obtained to ensure adequate sequencing depth. Consensus sequence generated in this study have been submitted to the NCBI database under accession number PV650320. Both Mycobank database-based identification and the constructed phylogenetic tree (Fig. 2) validated the species identifications, clustering the sample consistently within their respective species clades. In the literature, there is no information about isolation of an endophytic *Aspergillus westerdijkiae* from plant sources.

### Antimicrobial, antioxidant, and anticancer potentials

The pigment fractions 17P1, 17P2, 17P3 and 17P4 separated from the fungus *Aspergillus westerdijkiae* 17P were tested against prominent bacterial and fungal pathogens as depicted in Tables 1 and 2. The results indicate Zone of Inhibition (ZOI) and Minimum Inhibitory Concentration (MIC) values of pigments against the pathogens. All the fractions showed inhibitory activity against *E. coli* and *C. albicans*, while the fraction, 17P2, showed inhibition against all tested pathogens. Notably, 17P2 displayed ZOI at 100 mg/mL against *E. coli* and *S. aureus* and 250 mg/mL against *P. aeruginosa* and *K. pneumoniae*. With fungal pathogens, 17P2 displayed ZOI at 500 mg/mL against *A. brasiliensis*, *A. alternata* and *F. oxysporum* and 250 mg/mL against *C. albicans*. Inhibitory activity of 17P4 was observed at 500 mg/mL concentration against *E. coli* and *C. albicans* and at 1000 mg/mL against *F. oxysporum*. 17P1 showed activity against *E. coli* (250 mg/mL), *S. aureus* (250 mg/mL) and *C. albicans* (500 mg/mL) while 17P3 showed activity against *E. coli*,* S. aureus*,* A. alternata* and *C. albicans* at 500 mg/mL concentration. In the literature, data regarding antimicrobial potential of pigments from *Aspergillus westerdijkiae* are rare. Generally, the research on fungal endophytes have resulted in the discovery of several biologically active compounds [32]. Several of these metabolites exhibit potent antimicrobial properties, effectively inhibiting the growth of pathogenic bacteria and fungi [33].

**Table 1:** Antibacterial activity of the separated fractions from *Aspergillus westerdijikae* 17P cultures against different Gram-positive and Gram-negative human pathogenic bacterial strains

Separated fractions			Bacterial pathogens		
		*E. coli*	*S. aureus*	*P. aeruginosa*	*K. pneumoniae*
17P1	ZOI (mm)	9.67±0.88	10.43±0.91	Nil	Nil
	MIC (mg mL^-1^)	250	250	0.00	0.00
17P2	ZOI (mm)	11.43±0.87	10.78±0.54	9.08±0.67	11.78±0.76
	MIC (mg mL^-1^)	100	100	250	250
17P3	ZOI (mm)	8.67±0.56	9.35±0.34	Nil	Nil
	MIC (mg mL^-1^)	500	500	0.00	0.00
17P4	ZOI (mm)	10.48±0.98	Nil	10.39±0.76	Nil
	MIC (mg mL^-1^)	500	500	0.00	0.00
Amoxicillin/clavulanic acid		13.98±0.77	16.79±0.91	18.31±0.61	11.33±0.79
LSD		0.3981	0.4388	0.521	0.4511

Amoxicillin/clavulanic acid was used as the positive control at a concentration of 500 µg mL^− 1^. The calculated mean is for triplicate measurements from three independent experiments ± SD. Nil means that no ZOI was detected. LSD means Least Significant Differences

**Table 2:** Antifungal activity of the separated fractions from *Aspergillus westerdijikae* 17P cultures against different human and plant pathogenic fungi

Separated fractions		Fungal pathogens			
		*A. brasiliensis*	*A. alternata*	*F. oxysporum*	*C. albicans*
17P1	ZOI (mm)	Nil	Nil	Nil	14.43 ± 0.56
	MIC (mg mL^− 1^)	0.00	0.00	0.00	500
17P2	ZOI (mm)	10.38 ± 0.87	9.61 ± 0.33	11.87 ± 0.31	17.39 ± 0.65
	MIC (mg mL^− 1^)	500	500	500	250
17P3	ZOI (mm)	Nil	10.21 ± 0.33	0.00	12.34 ± 0.57
	MIC (mg mL^− 1^)	0.00	1000	0.00	1000
17P4	ZOI (mm)	Nil	Nil	10.31 ± 0.54	11.34 ± 0.32
	MIC (mg mL^− 1^)	0.00	0.00	1000	500
Nystatin		15.34 ± 0.67	12.41 ± 0.33	14.67 ± 0.34	15.97 ± 0.61
LSD		0.3187	0.1891	0.1012	0.2198

Nystatin was used as the positive control at a concentration of 500 µg mL^− 1^. The calculated mean is for triplicate measurements from three independent experiments ± SD. LSD means Least Significant Differences

Results of scavenging activity of the separated fractions showed that the activity increases with increasing concentration as stated in Table 3. When compared to 17P1 and 17P3, it can be observed that the fractions 17P2 and 17P4 show higher activity. Nearly 83% and 67% of free radicals were scavenged by fractions 17P2 and 17P4 respectively at a concentration of 1000 mg/mL, demonstrating the most promising antioxidant potential. Reports on the antioxidant potential of pigments produced by *Aspergillus westerdijkiae* remain scarce in the literature. In general, studies on fungal endophytes have led to the identification of numerous biologically active compounds [34], many of which display potent antioxidant properties by effectively scavenging reactive oxygen species.

**Table 3:** Antioxidant activity of the separated fractions from *Aspergillus westerdijikae* 17P cultures

Concentration (mg mL^− 1^)	Scavenging activity (%)			
	17P1	17P2	17P3	17P4
0.00	0.00	0.00	0.00	0.00
0.1	0.00	27.31 ± 10.78	0.00	10.43 ± 0.77
10	8.71 ± 2.79	44.29 ± 9.39	10.89 ± 1.89	28.71 ± 2.79
100	23.54 ± 10.98	61.66 ± 10.51	29.43 ± 11.43	43.54 ± 10.98
1000	47.66 ± 12.54	83.54 ± 11.32	55.43 ± 21.78	67.66 ± 12.54
LSD	3.62	4.667	3.62	4.667

Ascorbic acid (antioxidant standard) was the control. The calculated mean is for triplicate measurements from three independent experiments ± SD, LSD = least significant differences (*p* ≤ 0.05)

Cytotoxicity of the isolated pigments was tested against normal (Hfb-4), breast cancer (MCF-7) and liver cancer (HepG-2) cell lines (Table 4). Cell viability of 70–80% can be seen with fractions 17P1 and 17P3 at 500 mg/mL MIC against both cancer cell lines. At the MIC of 1000 mg/mL, 17P4 showed cell viability of 87% and 91% against breast and liver cancer cell lines. The pigment fraction 17P2 was considered more promising with a cell viability of 68% against MCF-7 and 48% against HepG-2 at a minimum inhibitory concentration of 250 mg/mL.

**Table 4:** Cytotoxic activities of the separated fractions from *Aspergillus westerdijikae* 17P cultures against normal, breast cancer, and liver cancer cell lines

		Cell line		
Separated fractions		Hfb-4	MCF-7	HepG-2
		(normal)	(breast)	(liver)
17P1	Cell viability (%)	66.52 ± 11.97	78.44 ± 14.83	70.09 ± 17.81
	MIC (mg mL^− 1^)	500	500	500
17P2	Cell viability (%)	57.83 ± 10.33	67.98 ± 12.67	48.78 ± 9.76
	MIC (mg mL^− 1^)	250	250	250
17P3	Cell viability (%)	84.76 ± 21.32	79.21 ± 17.43	80.32 ± 18.81
	MIC (mg mL^− 1^)	500	500	500
17P4	Cell viability (%)	90.55 ± 20.51	87.32 ± 15.45	91.45 ± 18.98
	MIC (mg mL^− 1^)	1000	1000	1000
Taxol		15.56 ± 1.33	17.08 ± 1.67	20.72 ± 1.87
LSD		5.839	8.728	7.451

Taxol was used as the positive control at a concentration of 50 µg mL^− 1^. MTT-based assay was used for measuring the cytotoxic activities at 570 nm using MTT solution under the conditions described in Materials and Methods. Calculated mean is for triplicate measurements from three independent experiments ± SD. LSD means Least Significant Differences

In the literature, endophytic fungi have attracted considerable scientific interest due to their ability to produce a wide range of bioactive secondary metabolites. To date, the processes of isolation, cultivation, purification, and characterization of these fungi have led to the identification of approximately 200 structurally diverse and biologically significant compounds, including metabolites with demonstrated antimicrobial [32, 33], antioxidant [34], and anticancer [35] activities.

### AChE, BChE and MAO-A inhibitory and PPAR-γ agonistic potential of the separated pigment fractions

ITC analysis allows for a better understanding of the interactions between the studied enzymes and the receptors and pigments. To determine binding affinity, an analysis was performed by measuring the heat during each injection of the ligand into the protein present in the cell. This allows to monitor heat changes until all bonds are saturated. The experiment also includes a reference test in which the ligand is injected into a reagent in which the sample has been diluted. This heat is subtracted from the ligand-protein experiment [36, 37]. The parameter indicated one protein binding site per ligand molecule [38]. The ITC technique allows the use of low enzyme/receptor concentrations, thus demonstrating affinity independent of biological effects, avoiding non-specific binding [39].

#### AChE and BChE

AChE were found to bind to 17P1 and 17P2, but not to bind to 17P3 and 17P4. Kd for 17P2 was 1.63 µmol/L, and for 17P1 it was 2.78 µmol/L (Table 5). The enthalpy, ΔS, showed a value of -31.09 J/mol*K for 17P1 and + 71.06 J/mol*K for 17P2, which means that the 17P2 compound showed a stronger interaction, confirming the high affinity between 17P2 and AChE (ΔG -13.28 kJ/mol). In the case of BChE, compounds 17P1, 17P2 and 17P3 showed strong binding to BChE. Only 17P4 showed no binding to the active site of the enzyme. The highest affinity, similarly to AChE, was shown by compound 17P2, with a value of -124.98 kJ/mol (Table 6), with a positive reaction enthalpy ΔH = 7.69 kJ/mol. The dissociation constant was comparable for 17P1 and 17P2, being 0.03 µmol/L, while compound 17P3 had a slightly lower value of 0.01 µmol/L. The IC50 for AChE showed the lowest ligand dose inhibiting 50% of the enzyme for 17P1, while for BChE it was the 17P1 compound. Fungi are known to produce a diverse array of bioactive metabolites capable of inhibiting acetylcholinesterase (AChE) and butyrylcholinesterase (BChE). The extent of cholinesterase inhibition by fungal extracts varies considerably; some exhibit potent AChE inhibition, while others are more selective for BChE [40]. This variability indicates the presence of multiple bioactive constituents within fungal species. Notably, out of 43 fungal strains screened, six isolates demonstrated differing levels of inhibitory activity were reported, underscoring the strain-specific nature of these effects [41].

**Table 5:** Thermodynamic parameters of interactions between AChE and the separated fractions from *Aspergillus westerdijikae* 17P cultures

Separated frctions	K_d_ (µmol L^− 1^)	K_a_ *10^3^ (L mol^− 1^)	∆H (kJ mol^− 1^)	∆G (kJ mol^− 1^)	∆S (J mol*K^− 1^)	Inhibitor activity [%]	IC_50_ (0.25 µL µM AChE i 10 µMAChE^− 1^ (%))	K_i_ (µmol L^− 1^) K_M_ ACh 35.91
17P1	2.78 ± 0.03^a^	0.10 ± 0.00 ^a^	10.00 ± 0.02 ^a^	35.11 ± 0.45 ^a^	-71.06 ± 0.21 ^a^	86.11 ± 0.15 ^a^	5.28 ± 0.02 ^a^	0.34 ± 0.01 ^a^
17P2	1.63 ± 0.02^b^	5.62 ± 0.05 ^b^	0.18 ± 0.01 ^b^	-13.28 ± 0.45 ^b^	-31.09 ± 0.98 ^b^	75.22 ± 0.18 ^b^	6.04 ± 0.04 ^b^	0.05 ± 0.00 ^b^
17P3	Nb	Nb	Nb	Nb	Nb	Nb	Nb	Nb
17P4	Nb	Nb	Nb	Nb	Nb	Nb	Nb	Nb

Values are expressed as mean ± SD; *n* = 4; different letters in one column correspond to significant differences (*P* < 0.05); Nb – no binding

**Table 6:** Thermodynamic parameters of interactions between BChE and the separated fractions from *Aspergillus westerdijikae* 17P cultures

Separated frctions	K_d_ (µmol L^− 1^)	K_a_ *10^3^ (L mol^− 1^)	∆H (kJ mol^− 1^)	∆G (kJ mol^− 1^)		∆S (J mol*K^− 1^)		Inhibitor activity [%]	IC_50_ (0,25 µL µM BChE i 10 µM BChE^− 1^ (%))	Ki (µmol L^− 1^) K_M_ BCh 35.91
17P1	0.03 ± 0.00^a^	25.40 ± 0.48^a^	0.03 ± 0.00^a^	-25.99 ± 0.78^a^		-27.21 ± 0.07^a^		46.86 ± 0.15^a^	12.33 ± 0.03^a^	0.01 ± 0.00^a^
17P2	0.03 ± 0.00^a^	25.13 ± 0.02^a^	7.69 ± 0.02^b^	-124.98 ± 0.52^b^		-40.75 ± 0.12^b^		99.37 ± 0.45^b^	4.57 ± 0.02^b^	0.27 ± 0.01^b^
17P3	0.02 ± 0.00^b^	18.30 ± 0.15^b^	0.12 ± 0.01^c^	-18.98 ± 0.19^c^		-28.04 ± 0.55^a^		62.23 ± 0.26^c^	7.29 ± 0.03^c^	0.02 ± 0.00^c^
17P4	Nb	Nb	Nb	Nb		Nb		Nb	Nb	Nb

Values are expressed as mean ± SD; *n* = 4; different letters in one column correspond to significant differences (*P* < 0.05); Nb – no binding

#### MAO-A

Obtained fractions bound at the active site. The results are presented in Table 7. The dissociation constant ranged from 0.13 to 1 µmol/L for 17P3 and 17P2, respectively. The highest interaction constant and affinity was shown by compound 17P2, which was ΔH = 100 kJ/mol and ΔG = 153 kJ/mol. On the other hand, the lowest interaction constant and the highest affinity were observed for compound 17P3, which was ΔH = 0.01 kJ/mol and ΔG= -47.23 kJ/mol. Fraction 17P3 also showed the lowest value needed to achieve half-maximal inhibitory effect, which was 4.03 µmol/µmol enzyme. Reports on metabolites with monoamine oxidase A (MAO-A) inhibitory activity from endophytic fungi remain scarce in the literature. Nevertheless, a few fungal species have been identified as sources of metabolites with MAO-A inhibitory potential. For instance, *Daldinia fissa* was found to produce 5-hydroxy-2-methylchroman-4-one, which exhibited dual inhibition of MAO-A and MAO-B [42]. In another study, (*R*)-5-methylmellein, isolated from the mycelial fermentation of *Xylaria nigripes*, acted as a selective MAO-A inhibitor and contributed to the understanding of its antidepressant mechanisms [43].

**Table 7:** Thermodynamic parameters of interactions between MAO-A and the separated fractions from *Aspergillus westerdijikae* 17P cultures

Separated frctions	K_d_ (µmol L^− 1^)	K_a_ *10^3^ (L mol^− 1^)	∆H (kJ mol^− 1^)	∆G (kJ mol^− 1^)	∆S (J mol*K^− 1^)	Inhibitor activity [%]	IC_50_ (1 µmol L inhibitor^− 1^: 1 µmol L^− 1^ MAO-A)	K_i_ (µmol L^− 1^) K_M_ 5-HT 0.34 mmol
17P1	0.24 ± 0.01^a^	0.472 ± 0.13^a^	2.11 ± 0.11^a^	2.64 ± 0.16^a^	-37.49 ± 0.33^a^	77.78 ± 0.44^a^	7.46 ± 0.13 ^a^	1.13 ± 0.05 ^a^
17P2	1.00 ± 0.00^b^	0.001 ± 0.00^b^	100.00 ± 1.18^b^	153.40 ± 0.15^b^	-71.06 ± 0.85^b^	81.44 ± 0.44^b^	6.55 ± 0.51^b^	0.84 ± 0.05^b^
17P3	0.13 ± 0.01^c^	118.00 ± 0.98^c^	0.01 ± 0.00^c^	-47.23 ± 0.09^c^	-17.34 ± 0.14^c^	97.05 ± 0.52^a^	4.03 ± 0.78^c^	0.88 ± 0.05^b^
17P4	0.35 ± 0.01^d^	5.82 ± 0.22^d^	0.17 ± 0.02^d^	9.94 ± 0.11^d^	-30.26 ± 0.44^d^	94.00 ± 0.58^c^	5.49 ± 0.15^b^	0.94 ± 0.02^d^

Values are expressed as mean ± SD; *n* = 4; different letters in one column correspond to significant differences (*P* < 0.05); Nb – no binding

#### PPAR-γ

The obtained fractions bind to the active site of PPAR-γ. The results are presented in Table 8. The dissociation constant is in the range of 7.13–8.70 µmol/L, for 17P1 and 17P3, respectively. The lowest reaction enthalpy is characterized by the 17P3 fraction, amounting to -19.65 kJ/mol, but it shows a slightly lower affinity for the receptor compared to the other fractions, amounting to -29.97 kJ/mol. The typical ligand, 15-deoxy-Δ12,14-prostaglandin J2, showed a higher affinity by about − 18 kJ/mol. Literature on peroxisome proliferator-activated receptor gamma (PPAR-γ) agonists from endophytic fungi is extremely limited. Three studies to date have reported relevant fungal metabolites. The first described the isolation of five novel compounds from *Cladosporium oxysporum* [44], while the second identified four new bioactive metabolites (dihydrotrichodimerol, trichodimerol, rezishanones C, and rezishanones D) through bioassay-guided fractionation of an unidentified fungal strain [45]. The third described six different endophytes with varying agnostic levels [41].

**Table 8:** Thermodynamic parameters of interactions between PPAR-γ and the separated fractions from *Aspergillus westerdijikae* 17P cultures

Separated fractions	*N*	K_d_ (µmol L^− 1^)	K_a_ *10^4^ (L mol^− 1^)	∆H (kJ mol^− 1^)	∆G (kJ mol^− 1^)	∆S (J mol*K^− 1^)
17P1	0.09 ± 0.01^a^	7.13 ± 0.03^a^	1.40 ± 0.05^a^	-16.72 ± 0.13^a^	-30.51 ± 0.15^a^	44.53 ± 0.38^a^
17P2	0.07 ± 0.01^b^	7.80 ± 0.01^a^	1.28 ± 0.01^b^	-11.70 ± 0.15^b^	-30.26 ± 0.13^a^	59.92 ± 0.16^b^
17P3	0.08 ± 0.00^b^	8.70 ± 0.02^b^	1.15 ± 0.01^c^	-19.65 ± 0.05^c^	-29.97 ± 0.28^b^	33.33 ± 0.27^c^
17P4	0.09 ± 0.01^a^	7.82 ± 0.02^a^	1.28 ± 0.02^a^	-10.45 ± 0.15^b^	-30.26 ± 0.11^a^	63.97 ± 0.21^d^
Ligand	0.99 ± 0.02^c^	0.05 ± 0.00^c^	189.75 ± 2.15^d^	-98.81 ± 0.95^b^	-48.99 ± 1.35^c^	-0.16 ± 0.01^e^

Values are expressed as mean ± SD; *n* = 4; different letters in one column correspond to significant differences (*P* < 0.05); Nb – no binding

### Effect of gamma irradiation on pigments production

Data presented in Table 9 indicate that exposure to low doses of gamma irradiation (250 and 500 Gy) resulted in spore survival rates of 99.48% and 89.48%, respectively. However, a marked decline in spore viability was observed at 2000 Gy, with survival reduced to 79.38%. At the highest applied dose (16,000 Gy), no fungal growth was detected, indicating a complete loss of viability. These findings suggest a dose-dependent inhibitory effect of gamma irradiation on fungal viability and growth. Correspondingly, dry biomass measurements showed that higher doses of gamma irradiation (4000 and 8000 Gy) led to the most pronounced reductions in biomass accumulation, with yields declining to 3.78 g/L and 1.08 g/L, respectively. This further supports the observation of dose-dependent suppression of fungal growth.

**Table 9:** Effect of gamma irradiation on survival rate (%), fungal growth (g L^**− 1**^), and yellow, orange and red pigments production by *Aspergillus westerdijikae* 17P

Dose (kGy)	Survival (%)	Dry biomass (g L^− 1^)	Pigment yield (AU g^− 1^ fresh biomass)		
			Red	Orange	Yellow
0.00	100.00 ± 0.00	10.76 ± 0.56	0.87 ± 0.02	0.54 ± 0.03	0.71 ± 0.09
0.25	99.48 ± 0.28	10.01 ± 0.87	1.01 ± 0.21	0.57 ± 0.01	0.73 ± 0.10
0.50	89.48 ± 1.88	9.43 ± 0.21	1.43 ± 0.35	0.79 ± 0.13	0.84 ± 0.15
1.00	80.78 ± 2.87	7.88 ± 1.03	1.47 ± 0.26	0.79 ± 0.21	1.06 ± 0.21
2.00	79.38 ± 6.87	5.34 ± 0.87	2.98 ± 0.81	1.19 ± 0.42	1.19 ± 0.41
4.00	39.44 ± 12.98	3.78 ± 0.98	1.81 ± 0.16	1.06 ± 0.55	0.97 ± 0.17
8.00	2.73 ± 1.02	1.08 ± 0.37	0.11 ± 0.01	0.37 ± 0.01	0.27 ± 0.11
16.00	0.00	0.00	0.00	0.00	0.00
LSD	9.1873	0.8661	0.3121	0.1198	0.2136

The concentration was measured at 410, 470, and 510 nm that corresponded to the characteristic absorbance of yellow, orange and red pigments, respectively. The calculated mean is for triplicate measurements from two independent experiments ± SD. LSD means Least Significant Differences

Regarding pigment biosynthesis, exposure to 2000 Gy significantly enhanced the production of yellow, orange, and red pigments by *A. westerdijkiae* 17P compared to the non-irradiated control. This dose was identified as optimal for stimulating pigment biosynthesis. However, further increases in irradiation dose (4000–8000 Gy) resulted in a progressive decline in pigment production across all three pigment classes. In the literature, gamma radiation, a potent form of ionizing energy, can induce substantial biological effects, primarily by generating mutations through DNA strand breaks and errors during subsequent repair processes [46, 47]. Mutagenesis induced by gamma rays has been widely applied in microbial biotechnology to improve strain performance [48–53]. This approach typically involves repeated exposure to physical or chemical mutagens to introduce random genetic variability, followed by screening for strains with enhanced biosynthetic traits [54]. Our findings demonstrate that gamma irradiation at a dose of 2000 Gy (2 kGy) can significantly enhance pigment production in *A. westerdijkiae*, potentially due to mutations induced within biosynthetic gene clusters responsible for secondary metabolite synthesis [55].

### Chemical characterization

**Fig. 3:**
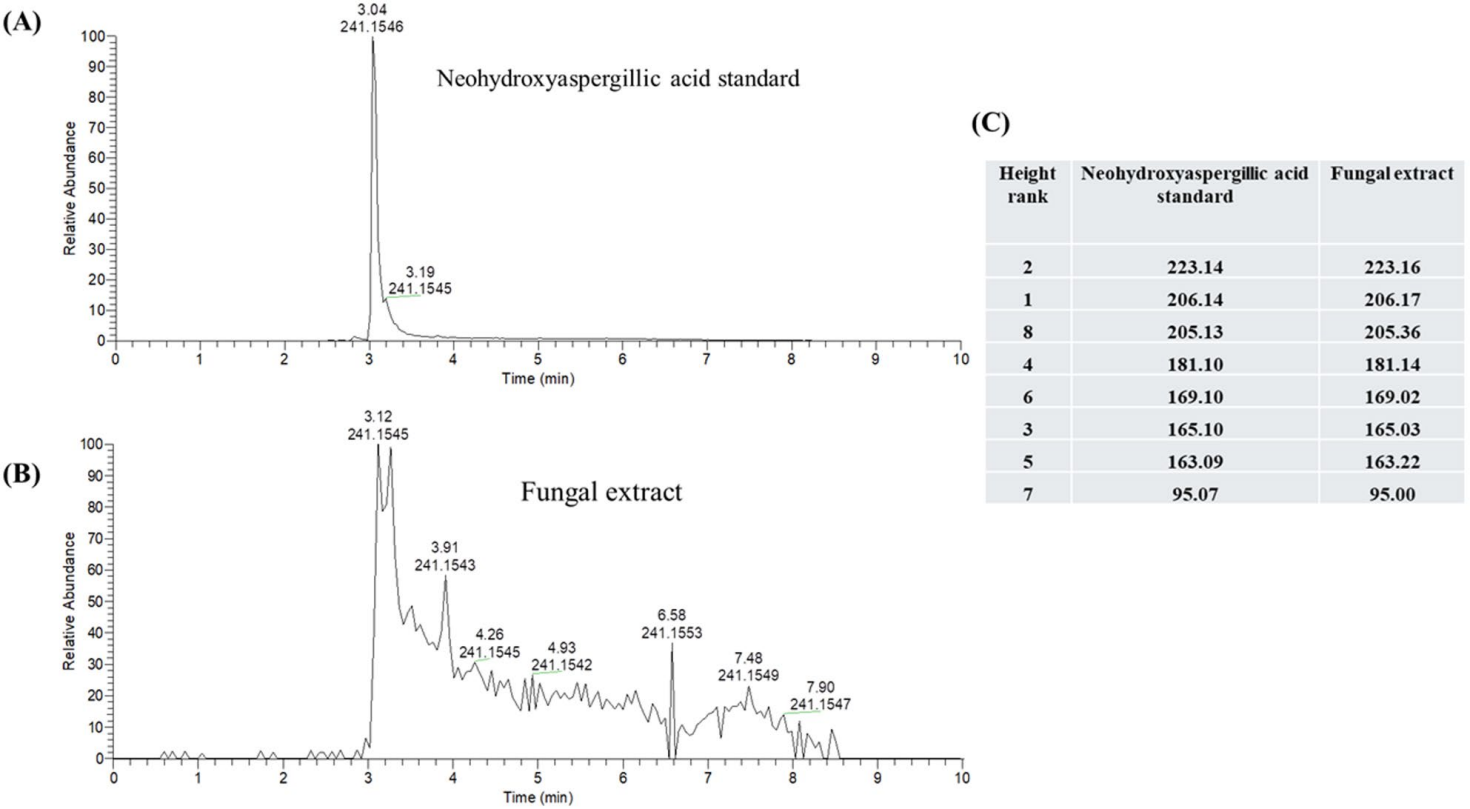
LC-MS dereplication analysis of *Aspergillus westerdijkiae* 17P extract. Panel **A** shows the elution time and HRMS measurement for the standard of neohydroxyaspergillic acid. Panel **B** shows the LC-MS chromatogram for extract, with the peak at 3.12 min correlating well with the above standard. Panel **C** shows the MS-MS fragmentation data for both the standard of neohydroxyaspergillic acid and the peak at 3.12 min in the extract, where there was excellent agreement between the top eight fragments

The whole biomass extract of the fungus was analyzed via LC-MS dereplication using a database of over 700 fungal metabolites [28, 29]. An [M + H]^+^ ion peak of m/z 241.1545 was observed in the positive mode at 3.12 min, which matched well with the standard for neohydroxyaspergillic acid and with a calculated [M + H]^+^ of m/z 241.1547 and an elution time of 3.04 min (Fig. 3). This assignment was further confirmed via MS-MS fragmentation, where there was excellent agreement between the top 8 fragments for both the standard and the peak within the context of the extract (Fig. 3C).

**Fig. 4:**
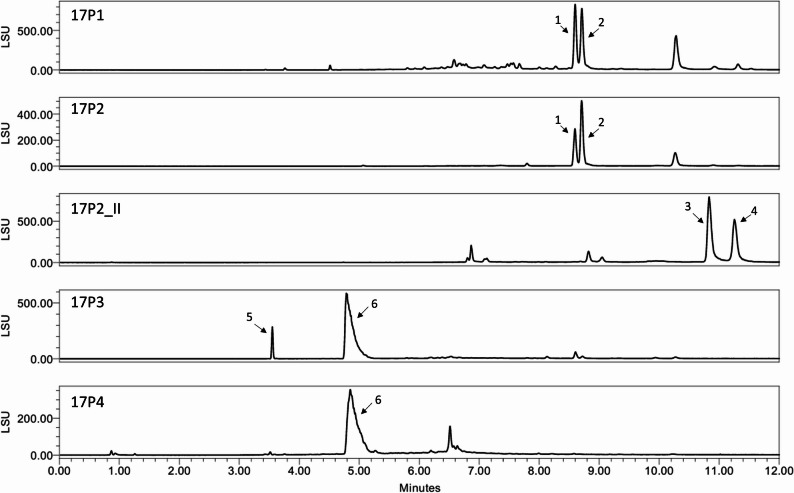
UHPLC-ELSD chromatograms of the separated fractions from the biomass extract of the *Aspergillus westerdijkiae* 17P

To gain more insight into the chemical profile of the investigated fractions (17P1-17P4), UHPLC-ELSD and UHPLC-UV-MS analyses were performed, Table 10). The ELSD chromatograms (Fig. 4) revealed a similar chemical composition for the fraction pairs 17P1 and 17P2 as well as for 17P3 and 17P4. To determine the constituents contributing to the shiny orange color of fractions 17P1 and 17P2, size exclusion chromatography was conducted which resulted in five fractions (17P2_I-V), with 17P2_II containing the orange-colored pigments. UHPLC-UV-MS analysis of this fraction enabled the tentative annotation of two constituents (**3**,** 4**) as metal complexes, bearing either iron or aluminum as central metal ions each forming coordinate bonds with three units of aspergillic acids or its isomer, neoaspergillic acid. Such pigments have been previously described for other *Aspergillus* species [56]. The two main constituents (**1**, **2**; both *m/z* 499.32 in neg. mode) of 17P1 and 17P2 were found in the sub-fractions 17P2_III and 17P2_IV in mixture with other constituents (not shown). Compounds **5** and **6** present in fractions 17P3 and 17P4 were tentatively annotated as penicillic acid (*m/z* 171.12 [M + H]^+^) and preussin (*m/z* 318.27 [M + H]^+^) based on UHPLC-MS, respectively. These annotations were confirmed by 1D and 2D NMR analysis of fraction 17P4, with the detected signals aligned well with previously reported NMR data for compound **5** [57] and **6** [58, 59]. Since compound **6** was in a mixture, its absolute configuration could not be determined.

**Table 10:** Structural annotation of detected compounds in fractions 17P1-17P4 and 17P2_II

Peak no.	RT [min]	UV max [nm]	m/z [pos]	m/z [neg]	Structure annotation
1	8.59	n. d.	n. d.	499.3	Unknown
2	8.71	n. d.	n. d.	499.4	Unknown
3	10.83	316	697.5 [M + H]^+^, 719.5 [M + Na]^+^, 735.5 [M + K]^+^	n. d.	Aspergillic acid aluminium complex
4	11.25	309	726.5 [M + H]^+^, 748.5 [M + Na]^+^, 764.5 [M + K]^+^	n. d.	Aspergillic acid iron complex
5	3.55	n. d.	371.13 [M + H]^+^	n. d.	Penicillic acid*
6	4.79	n. d.	318.27 [M + H]^+^	n. d.	Preussin*

n. d., not detected; *, Identified by NMR

## Conclusion

*Aspergillus westerdijkiae* 17P, isolated from *Betula pendula*, demonstrates strong potential as a sustainable source of multifunctional fungal pigments. Fraction 17P2 exhibited notable antimicrobial, antioxidant, anticancer, neuroprotective activities, and metabolic regulatory PPAR-γ agonist potentials. Structural analysis revealed key bioactive metabolites, and pigment production was significantly enhanced by gamma irradiation. These findings highlight the strain’s promise for pharmaceutical and industrial applications of natural biopigments. Since we have identified aluminum- and iron-complexed pigment fractions, we hypothesize that the metal coordination may enhance the stability of the pigment, modulate its redox behaviour (for antioxidant activity), or influence its interaction with microbial/enzymatic targets (for antimicrobial activity). Future work (e.g., comparative assays of metal-coordinated vs. de-metalated pigments) is planned to test this. Moreover, the industrial potential of these fungal pigments could be realized through scaling up production via optimized fermentation and efficient downstream processing.

## Data Availability

All data generated or analyzed during this study are included in this published article.
