# Correction: Natural pigments from the endophyte *Aspergillus westerdijkiae* and evaluation of their bioactivities

**DOI:** 10.1186/s12934-026-02958-3

**Published:** 2026-02-19

**Authors:** Abirami Baskaran, Andreas Wasilewicz, Judith M. Rollinger, Joanna Grzelczyk, Ilona Gałązka-Czarnecka, Grażyna Budryn, Tomasz Strzała, Tyler N. Graf, Nicholas H. Oberlies, Filip Boratyński, El-Sayed R. El-Sayed

**Affiliations:** 1 Department of Food Chemistry and Biocatalysis, Wrocław University of Environmental and Life Sciences, Norwida 25, Wrocław, 50-375 Poland; 2 Division of PharmacognosyDepartment of Pharmaceutical Sciences, University of Vienna, Josef-Holaubek-Platz 2, Vienna, 1090 Austria; 3 Institute of Food Technology and AnalysisFaculty of Biotechnology and Food Sciences, Lodz University of Technology, Lodz, 90-537 Poland; 4 Department of Genetics, Wroclaw University of Environmental and Life Sciences, Kożuchowska 7, Wrocław, 51-631 Poland; 5 Department of Chemistry and Biochemistry, University of North Carolina at Greensboro, Greensboro, NC 27402 USA; 6 Plant Research DepartmentNuclear Research Center, Egyptian Atomic Energy Authority, Cairo, Egypt

**Correction: Microbial Cell Factories (2026) 25:32**

 10.1186/s12934-025-02905-8.

In this article [1], the errors occurred in Tables 1, 2 and 4. Also, some text corrections such as antifungal and antibacterial name have been updated.

For completeness and transparency, the incorrect and corrected tables, texts are displayed below.

The original article has been corrected. 

## Correction 1

In heading “Materials and methods” under sub heading “Antimicrobial activity” the second sentence in the paragraph has been corrected. 

### Incorrect Sentence:

Antibacterial efficacy was evaluated against human pathogenic strains Staphylococcus aureus (ATCC 6538) and Escherichia coli (ATCC 11229), while antifungal activity was tested against Fusarium oxysporum (EUM37, a phytopathogen), Candida albicans (ATCC 10231), and Aspergillus brasiliensis (ATCC 16404). 

### Corrected Sentence:

Antibacterial efficacy was evaluated against human pathogenic strains, including *Staphylococcus aureus* ATCC6538, *Pseudomonas aeruginosa* ATCC9027, *Klebsiella pneumoniae* ATCC13883, and *Escherichia coli* ATCC11229, while antifungal activity was tested against *Fusarium oxysporum* EUM37, *Alternaria alternata* EUM108 (phytopathogens), *Candida albicans* ATCC10231, and *Aspergillus brasiliensis* ATCC16404 (human pathogens).

## Correction 2

In the section “Materials and methods”, under the sub heading “Cytotoxic activity assay” the term “melanoma (A375)” appearing in the third line should be deleted.

### Incorrect sentence: 

The cytotoxic effects of pigment fractions (17P1–17P4) were evaluated against three human cancer cell lines: melanoma (A375), breast adenocarcinoma (MCF-7), and lung carcinoma (A549), along with a non-cancerous human fibroblast cell line (Hfb-4).

### Corrected sentence: 

The cytotoxic effects of pigment fractions (17P1–17P4) were evaluated against three human cancer cell lines: breast adenocarcinoma (MCF-7), and lung carcinoma (A549), along with a non-cancerous human fibroblast cell line (Hfb-4).

## Correction 3

In the section “Materials and methods”, under the sub heading “Chemical characterization” in second paragraph, the text “from 5% -98% B in 5.0,” has been corrected to “5% − 98% B in 5 min” in the third sentence.

### Incorrect sentence: 

The column temperature was set to 40 °C, flow rate of 0.3 was used and the following gradient was applied: 5% B at 0.0 min, from 5% − 98% B in 5.0, 98% B for 10 min.

### Corrected sentence: 

The column temperature was set to 40 °C, flow rate of 0.3 was used and the following gradient was applied: 5% B at 0.0 min, from 5% − 98% B in 5 min, 98% B for 10 min.

## Correction 4

In Table 1, the second column header “E.coli” has been corrected to “S. aureus”.

## Correction 5:

**Incorrect Table 2:**

**Table 2:** Antifungal activity of the separated fractions from *Aspergillus Westerdijikae* 17P cultures against different human and plant pathogenic fungi

Separated fractions		Fungal pathogens			
		*A. brasiliensis*	*A. alternata*	*F. oxysporum*	*C. albicans*
17P1	ZOI (mm)	Nil	Nil	Nil	14.43 ± 0.56
	MIC (mg mL^− 1^)	0	0	0	500
17P2	ZOI (mm)	10.38 ± 0.87	9.61 ± 0.33	11.87 ± 0.31	17.39 ± 0.65
	MIC (mg mL^− 1^)	500	500	500	250
17P3	ZOI (mm)	Nil	10.21 ± 0.33	0	12.34 ± 0.57
	MIC (mg mL^− 1^)	0	1000	0	1000
17P4	ZOI (mm)	Nil	Nil	10.31 ± 0.54	11.34 ± 0.32
	MIC (mg mL^− 1^)	0	0	1000	500

Nystatin was used as the positive control at a concentration of 500 µg mL^− 1^. The calculated mean is for triplicate measurements from three independent experiments ± SD. Nil means that no ZOI was detected. LSD means Least Significant Differences

**Correct Table 2:**

**Table 2:** Antifungal activity of the separated fractions from *Aspergillus Westerdijikae* 17P cultures against different human and plant pathogenic fungi

Separated fractions		Fungal pathogens			
		*A. brasiliensis*	*A. alternata*	*F. oxysporum*	*C. albicans*
17P1	ZOI (mm)	Nil	Nil	Nil	14.43 ± 0.56
	MIC (mg mL^− 1^)	0	0	0	500
17P2	ZOI (mm)	10.38 ± 0.87	9.61 ± 0.33	11.87 ± 0.31	17.39 ± 0.65
	MIC (mg mL^− 1^)	500	500	500	250
17P3	ZOI (mm)	Nil	10.21 ± 0.33	0	12.34 ± 0.57
	MIC (mg mL^− 1^)	0	1000	0	1000
17P4	ZOI (mm)	Nil	Nil	10.31 ± 0.54	11.34 ± 0.32
	MIC (mg mL^− 1^)	0	0	1000	500
Nystatin		15.34 ± 0.67	12.41 ± 0.33	14.67 ± 0.34	15.97 ± 0.61
LSD		0.3187	0.1891	0.1012	0.2198

Nystatin was used as the positive control at a concentration of 500 µg mL− 1. The calculated mean is for triplicate measurements from three independent experiments ± SD. Nil means that no ZOI was detected. LSD means Least Significant Differences

## Correction 6

In Table 4, the sentence “Nil means that no ZOI was detected” in the footnote should have been removed.

### Corrected Footnote

Taxol was used as the positive control at a concentration of 50 μg mL− 1. MTTbased assay was used for measuring the cytotoxic activities at 570 nm using MTT solution under the conditions described in Materials and Methods. Calculated mean is for triplicate measurements from three independent experiments ± SD. LSD means Least Significant Differences.
